# Discriminant and concurrent validity of a simplified DSM-based structured diagnostic instrument for the assessment of autism spectrum disorders in youth and young adults

**DOI:** 10.1186/1471-244X-11-204

**Published:** 2011-12-30

**Authors:** Gagan Joshi, Carter R Petty, Ronna Fried, Janet Wozniak, Jamie A Micco, Aude Henin, Robert Doyle, Maribel Galdo, Meghan Kotarski, Janet Caruso, Benjamin Meller, Stephen V Faraone, Joseph Biederman

**Affiliations:** 1 Clinical and Research Program in Pediatric Psychopharmacology, Massachusetts General Hospital, Boston, MA, USA; 2 Department of Psychiatry, Harvard Medical School, Boston, MA, USA; 3 Department of Psychiatry, SUNY Upstate Medical University, Syracuse, NY, USA

## Abstract

**Background:**

To evaluate the concurrent and discriminant validity of a brief DSM-based structured diagnostic interview for referred individuals with autism spectrum disorders (ASDs).

**Methods:**

To test concurrent validity, we assessed the structured interview's agreement in 123 youth with the expert clinician assessment and the Social Responsiveness Scale (SRS). Discriminant validity was examined using 1563 clinic-referred youth.

**Results:**

The structured diagnostic interview and SRS were highly sensitive indicators of the expert clinician assessment. Equally strong was the agreement between the structured interview and SRS. We found evidence for high specificity for the structured interview.

**Conclusions:**

A simplified DSM-based ASD structured diagnostic interview could serve as a useful diagnostic aid in the assessment of subjects with ASDs in clinical and research settings.

## Background

Autism spectrum disorders (ASDs) comprise a group of neuropsychiatric disorders that include autistic disorder, Asperger's disorder, and pervasive developmental disorder not otherwise specified (PDD-NOS). They are distinguished from other psychiatric disorders by the presence of deficits in reciprocal social behavior, variously accompanied by deficits in communication, and/or repetitive or stereotyped behaviors. The DSM-III-R [1] and DSM-IV [2] have operationalized the required diagnostic criteria necessary for establishing diagnoses of ASDs based on the presence or absence of a set of categorical symptoms.

While a thorough evaluation by an expert clinician who has significant experience in specific diagnoses is considered the best method of diagnosing complex conditions such as ASDs, structured diagnostic interviews have been developed to help non-expert clinicians elicit the required information for these diagnoses. The most widely used structured interview tool for establishing a diagnosis of autism in the research setting is the Autism Diagnostic Interview-Revised (ADI-R). This interviewer requires specelized training in order to administer it, and the training to become proficient in its administration is expensive and time consuming. Additionally, the ADI-R takes at least 2 hours to complete making it of limited feasibility in clinical settings and in large population-based studies.

In contrast to the 93 questions and associated complex algorithms of the ADI-R, the DSM includes only 16 items in its diagnostic criteria for ASDs. Moreover, because the literature clearly indicates that most individuals with ASD have other comorbid conditions such as Attention-Defecit/Hyperactivity Disorder (ADHD) and mood disorders [3-7] and the ADI-R by itself does not assess other disorders that are pervasive in this population, another means for diagnosing other conditions is necessary and increases the length of time needed for a full assessment. This situation calls for the development of simplified instruments to aid in the assessment of ASDs in clinical and non-clinical settings.

Several attempts have been made to simplify the complexity of the assessment process for youth with ASDs. One such effort is development of the Social Responsiveness Scale (SRS), a paper and pencil instrument that can be completed by parents or teachers in 15-20 minutes. Constantino and colleagues [8,12] demonstrated its concurrent and discriminant validity as a measure of ASDs. As part of these efforts, Constantino et al. [8] compared the SRS with the ADI-R in 61 child psychiatric patients. Correlations between SRS scores and ADI-R algorithm scores for DSM-IV criterion sets were on the order of 0.7. SRS scores were unrelated to I.Q. and exhibited inter-rater reliability on the order of 0.8. Though SRS is a valid quantitative measure of autistic traits, the instrument lacks the ability to distinguish autism from the spectrum (Asperger's disorder and PDD-NOS) in individuals with ASD [8]. Despite the utility of the SRS as a screening tool for ASD, there continues to be a need to have a simplified DSM-based structured diagnostic interview module to aid in the diagnosis of individuals with ASD in clinical and research settings.

The main aim of the present study was to evaluate the concurrent and discriminant validity of a simplified, relatively brief, structured, diagnostic interview closely linked to the defining features of ASDs in the DSM (see Table 1). To examine the concurrent validity of this instrument, we examined its correspondence with a gold standard expert clinician's diagnoses in a large sample of clinically referred youth with ASDs. In addition, we examined its correspondence with the SRS because of the previously documented excellent correspondence between the SRS and the ADI-R [8]. To examine the discriminant validity of the DSM-ASD structured diagnostic interview, we calculated its specificity comparing subjects with ASD with those from a large sample of clinic referred youth with ADHD. We hypothesized that our DSM-based structured diagnostic interview for ASDs would have good concurrent and discriminant validity.

**Table 1:** ASD symptoms for the structured interview, DSM-III-R and DSM-IV

ASD structured interview	DSM-III-R Autistic Disorder	DSM-IV Autistic Disorder (Bold=Asperger's Disorder)
A. THESE ARE SOME QUESTIONS ABOUT HOW YOUR SON/DAUGHTER RELATES TO OTHERS.	A. QUALITATIVE IMPAIRMENT IN RECIPROCAL SOCIAL INTERACTION	**(1). QUALITATIVE IMPAIRMENT IN SOCIAL INTERACTION**
1. Did s/he seem unusually unaware of the existence or feelings of others?	1. Marked lack of awareness of the existence of or feelings of others	**(d) Lack of social or emotional reciprocity**
2. Did s/he not come for comfort even when hurt, or did s/he seek comfort in an odd way	2. No or abnormal seeking of comfort at times of distress	
3. Was s/he unable to imitate others when appropriate?	3. No or impaired imitation	
4. Does s/he have difficulty playing cooperatively with other children?	4. No or abnormal social play	**(c) A lack of spontaneous seeking to share enjoyment, interests, or achievements with other people**
5. Is s/he uninterested in making peer friendships? Or if s/he is interested, does s/he seem to understand the conventions of social interaction?	5. Gross impairment in ability to make peer friendships	**(b) Failure to develop peer relationships appropriate to developmental level**

B. THESE ARE SOME QUESTIONS ABOUT HOW YOUR SON/DAUGHTER COMMUNICATES OR PLAYS WITH OTHERS.	B. QUALITATIVE IMPAIRMENT IN VERBAL AND NONVERBAL COMMUNICATION, AND IN IMAGINATIVE ACTIVITY	(2). QUALITATIVE IMPAIRMENTS IN COMMUNICATION
1. Is s/he unable to communicate?	1. No mode of communication, such as communicative babbling, facial expression, gesture, mime, or spoken language	(a) Delay in, or total lack of, the development of spoken language (not accompanied by an attempt to compensate through alternative modes of communication such as gesture or mime)
2. Does s/he avoid looking at people or avoid greeting people? *If no, ask: *Does s/he ignore people around her/him, dislike being held?	2. Markedly abnormal nonverbal communication, as in the use of eye-to-eye gaze, facial expression, body posture, or gestures to initiate or modulate social interaction	**(1) (a) Marked impairment in the use of multiple nonverbal behaviors such as eye-to-eye gaze, facial expression, body postures, and gestures to regulate social interaction**
3. Is s/he uninterested in imaginative activities or stories?	3. Absence of imaginative activity, such as playacting of adult roles, fantasy characters, or animals; lack of interest in stories about imaginary events	(d) Lack of varied, spontaneous make-believe play or social imitative play appropriate to developmental level
4. When s/he speaks does her/his tone seem odd?	4, Marked abnormalities in the production of speech, including volume, pitch, stress, rate, rhythm, and intonation	
5. Did s/he repeat words or phrases s/he has just heard, in place of responding to what was said? Did s/he often use the wrong pronouns to refer to her/himself or others, or refer to him/herself in the third person, as "he wants a remarks cracker?"	5. Marked abnormalities in the form or content of speech, including stereotyped and repetitive use of speech; use of "you" when "I" is meant; idiosyncratic use of words or phrases; or frequent irrelevant remarks	(c) Stereotyped and repetitive use of language or idiosyncratic language
6. Did s/he seldom, if ever, start a conversation with someone else, even if s/he might talk to her/himself?	6. Marked impairment in the ability to initiate or sustain a conversation with others, despite adequate speech	(b) In individuals with adequate speech, marked impairment in the ability to initiate or sustain a conversation with others

C. THESE ARE QUESTIONS ABOUT SON/DAUGHTER' S ACTIVITIES OR INTERESTS	C. MARKEDLY RESTRICTED REPERTOIRE OF ACTIVITIES AND INTERESTS	**(3) RESTRICTED REPETITIVE AND STEREOTYPED PATTERNS OF BEHAVIOR, INTERESTS, AND ACTIVITIES**
1. Did s/he ever have any repetitive patterns of behavior such as hand movements, clapping or twirling?	1. Stereotyped body movements, e.g., hand-flicking or -twisting, spinning, head-banging, complex whole-body movements	**(c) Stereotyped and repetitive motor mannerisms**
2. Did s/he ever have any prolonged attachments to certain objects, either holding them or staring at them, or lining them up in a repetitive pattern?	2. Persistent preoccupation with parts of objects or attachment to unusual objects	**(d) Persistent preoccupation with parts of objects**
3. Did s/he ever get unusually upset if there were even small changes in where things were placed in the house?	3. Marked distress over changes in trivial aspects of environment	
4. Or get upset when there are changes in daily routine?	4. Unreasonable insistence on following routines in precise detail	**(b) Apparently inflexible adherence to specific, nonfunctional routines or rituals**
5. Does s/he have an extremely restricted range of interests, or a preoccupation with one very narrow interest	5. Markedly restricted range of interests and a preoccupation with one narrow interest	**(a) Encompassing preoccupation with one or more stereotyped and restricted patterns of interest that is abnormal either in intensity or focus**

## Methods

### Participants

ASD subjects were youth, ages 4 to 23 years of age, consecutively referred to a specialized program for the treatment of ASDs at a university-affiliated hospital. The diagnosis of ASD was established by a comprehensive psychiatric evaluation conducted by an board-certified psychiatrist experienced in evaluating ASD (GJ). The psychiatric diagnostic interview was conducted with the subject and caretaker (usually parent/s) and incorporated information from multiple sources when available (psychiatric records, schools, social services). Based on this clinical evaluation, all ASD subjects met Diagnostic and Statistical Manual of Mental Disorders - Fourth Edition (DSM-IV) diagnostic criteria for autistic disorder, Asperger's disorder, or PDD-NOS.

Psychiatric comparison participants were derived from consecutive referrals to a pediatric psychopharmacology program at a major academic center from 1991 to 2008. Children were referred for psychiatric evaluation and psychopharmacological intervention for behavioral and emotional difficulties and not for evaluation of any specific disorder. There was no selection bias based on social class or insurance restrictions. We included subjects if they met diagnostic criteria for ADHD on a structured diagnostic interview (Schedule for Affective Disorder and Schizophrenia for School-Age Children Epidemiologic Version [K-SADS-E]) [9]. The status of ASD in the ADHD control participants was assessed by the DSM based structured interview for ASD (see below). The structured diagnostic interview was completed by highly trained and supervised psychometricians from interviews with the parent, usually the mother. We computed kappa coefficients of agreement between these raters and experienced board certified child and adult psychiatrists and licensed clinical psychologists. Based on 500 assessments, the median kappa coefficient was 0.98. Kappa coefficients for ADHD was 0.88. Before final diagnostic assignments were made, information derived from these interviews was reviewed blindly by a committee of expert clinicians that included board certified child and adult psychiatrists and experienced licensed psychologists. We estimated the reliability of the diagnostic review process by computing kappa coefficients of agreement for clinician reviewers. For these diagnoses, the median reliability between individual clinicians and the review committee assigned diagnoses was 0.87. Kappa coefficients for ADHD was 1.0.

### Materials

For each ASD subject, a parent also completed The Social Responsiveness Scale (SRS)[10] a 65 item rating scale that measures the severity of autism spectrum symptoms including elements of reciprocal social behaviors (39 items), social use of language (6 items), and behaviors characteristic of children with autism spectrum disorders (20 items). Each item on the scale is rated on a Likert scale from "0" (never true) to "3" (almost always true). The psychometric properties of the SRS have been well established [11-14].

#### DSM-based structured interview for ASD

To assess ASDs we developed DSM-based diagnostic criterion into an interview format (see Table 1) using the parent as the informant. Diagnostic assessment of ASDs by this interview required a lifelong severe and pervasive deficit in development of reciprocal social interaction, communication, and restricted patterns of behavior. ASD subjects were defined as subjects meeting criteria for autistic disorder or PDD-NOS. To be given the diagnosis of autistic disorder, the participant had to meet DSM-III-R diagnostic criteria of eight out of sixteen symptoms with at least two symptoms from each of the three aforementioned domains of PDD. A diagnosis of PDD-NOS was received if more than two of the required symptoms were met with at least one symptom present from each of the three domains of PDD. This DSM-III-R based structured interview for ASD was developed prior to the release of DSM-IV criterion for ASD. This interview for ASD was administered to all the participants in this study. As data collection for the psychiatric comparison group in this study preceded the advent of DSM-IV, in order to maintain consistency in assessment the DSM-III-R criterion was retained beyond the release of DSM-IV. This DSM-based structured interview for ASD is added as a module to K-SADS-E and is administered by the trained interviewer in similar manner as the structured interview. All questions in the structured interview are asked in yes/no format. If the interviewee positively endorses a question, interviewers have specific follow-up questions they are required to ask. These questions include ages a symptom began/ended, if such statements have been true in the past month, and specific examples to elaborate on responses to the initial probes. Responses to follow-up questions help to determine whether each criterion is met. Supporting the interrater reliability of this diagnostic interview, the kappa coefficient of agreement for ASDs between the raters and experienced board certified child and adult psychiatrists and licensed clinical psychologists was 0.90. For ASDs, the reliability between an individual clinician and the review committee assigned diagnoses was 0.88. Table 1 also addresses the correlation between DSM-III-R criteria and DSM-IV criteria that clearly indicate that our DSM-based interview sufficiently covers both versions of the DSM.

#### Full scale IQ

Subjects with ASDs were assessed with the WASI [15]. Psychiatric comparison subjects were assessed with the WISC-R (N = 464) [16], WISC-3 (N = 97) [17], or the WASI (N = 77) [15]. The study was approved by the Institutional Review Board and in all cases parents gave written informed consent for participation.

### Statistical Approach

Conditional probabilities were calculated by determining how many clinically diagnosed ASD subjects and psychiatric comparison subjects met ASD criteria using the structured interview ASD module. We also determined how many ASD subjects had SRS scores of 60 or above. Then we examined the relationship between the structured interview diagnosis of ASD and scores on the SRS using two-sample t-tests. We randomly excluded 30% of the females from the original pool of the psychiatric comparison group so that both groups had similar gender ratios.

## Results

Out of 196 consecutive referrals to the program between October 2007 and August 2009, 123 individuals met the diagnostic criteria for ASD (75 autistic disorder, 22 Asperger's disorder, and 26 PDD-NOS) on clinical evaluation by the expert clinician (GJ). The ADHD psychiatric comparison group (ADHD group, N = 1563) did not significantly differ on age, sex, or ethnicity compared to the ASD group (Table 2). The ASD group had, on average, seven points lower full scale IQ compared to the ADHD group (p < 0.001, Table 2).

**Table 2:** Demographics and IQ scores

	**ASD (N = 123)**	**ADHD (N = 1563)**	**Test Statistic**	**p-value**
	
Age	10.9 ± 4.3	10.3 ± 3.3	t_(1684)_=1.89	0.06
% Male	109 (87)	1305 (83)	χ^2 ^_(1)_=0.60	0.44
% Caucasian	112 (89)	1198^a ^(93)	χ^2 ^_(1)_=2.57	0.11
Full Scale IQ	96.7 ± 18.4^b^	103.9 ± 15.1^c^	t_(695)_=3.38	<0.001

^a^out of N = 1284; ^b^N = 57; ^c^N = 640

### Concurrent Validity

**Figure 1:**
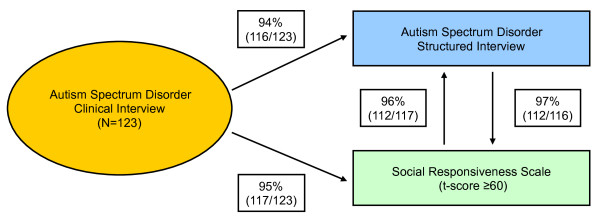
**Summary of agreement between the clinical interview, DSM-based autism spectrum structured interview, and Social Responsiveness Scale**.

Ninety-four percent (116/123) of the clinically diagnosed subjects with ASD also met criteria for ASD on the DSM-based structured diagnostic interview for ASDs resulting in a sensitivity of the DSM-based structured diagnostic interview for ASDs of 94%. Ninety-five percent (n = 117) of the clinically diagnosed subjects with ASD had an SRS t-score of 60 or higher (in the clinical range for ASD). Of the 116 subjects with a positive diagnosis of ASD on the DSM-based structured interview for ASD, 112 (97%) also had an SRS t-score of 60 or higher. Of the 117 subjects with an SRS t-score of 60 or higher, 112 (96%) had a positive diagnosis of ASD on the DSM-based structured interview diagnosis for ASD. Figure 1 summarizes the 3-way agreement found between clinical interview, the structured interview, and the SRS.

**Figure 2:**
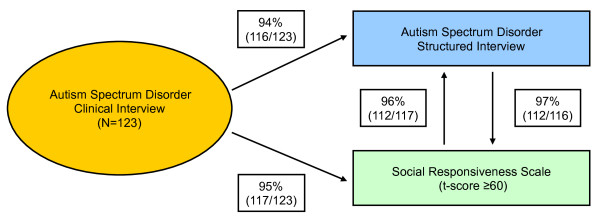
**A. Mean Social Responsiveness Scale (SRS) t-scores by structured interview autism spectrum disorder (ASD) diagnosis**. B. Percent with abnormal (≥ 60) Social Responsiveness Scale (SRS) t-scores by structured interview autism spectrum disorder (ASD) diagnosis.

Figure 2A shows the correspondence between the DSM-based ASD structured interview diagnosis and the SRS t-scores. The small number (n = 7) of subjects that did not meet criteria for ASD on the DSM-based structured interview for ASD (but met criteria for ASD by the expert clinician assessment) had a mean SRS t-score in the clinical range (t = 65.0, SD = 10.3) that was significantly lower than the mean SRS t-score in subjects with a structured diagnostic interview for ASDs corresponding to PDD-NOS (t = 77.5, SD = 11.7) and subjects with an ASD structured interview diagnosis of autistic disorder (t = 83.8, SD = 7.8). As shown in Figure 2B, subjects diagnosed with autistic disorder had a significantly higher rate of abnormal SRS scores compared to the other two groups.

### Discriminant Validity

Eleven percent (172/1563) of the ADHD group met structured interview criteria for ASDs on the DSM-based structured interview for ASDs. Therefore, a conservative estimate of the specificity of the DSM-based structured interview for ASD was 89%. Positive predictive value was 40% (116/288), and negative predictive value was 99.8% (1391/1398). In the ADHD group, subjects with a positive ASD diagnosis from the DSM-based structured interview were significantly younger than subjects without an ASD diagnosis (9.6 ± 3.5 versus 10.4 ± 3.3, z = -2.92, p = 0.003). Age was not significantly associated with the DSM-based structured interview diagnosis of ASD in the ASD group (z = 0.05, p = 0.96). In both of the groups, there was no relationship between meeting criteria for a structured interview diagnosis of ASDs and full scale IQ (ASD, z = 0.60, p = 0.54; ADHD, z = 0.51, p = 0.61). In the ASD group, subjects with a previous diagnosis of ASD were significantly more likely to receive a structured interview diagnosis of ASDs compared to subjects who not been previously diagnosed with ASD (previously diagnosed = 109/112, 97%; not previously diagnosed = 7/11, 64%; χ2(1) = 21.18, p < 0.001). Likewise, the clinician diagnosis was significantly associated with the structured interview diagnosis of ASD (autism = 100% received structured interview diagnosis of ASD; Asperger's disorder = 91%; PDD-NOS = 81%; χ2(2) = 13.88, p = 0.001).

## Discussion

The main purpose of the present study has been to evaluate the concurrent and discriminant validity of a simplified DSM-based structured diagnostic interview for the assessment of ASDs in a clinical setting. The present study reports excellent agreement of the DSM-based ASD structured diagnostic instrument with a gold standard expert clinician diagnosis of ASD based on a DSM-IV clinical assessment through detailed interviews with the patient and the parent. Results also showed excellent sensitivity and specificity when comparisons were made between subjects with ASD and subjects with ADHD. These results indicate that our DSM-based structured diagnostic interview for ASD can be a useful and cost-effective standardized assessment instrument for reliably identifying ASD in clinical and research settings. Many clinics and research settings employ diagnostic structured interviews for screening a broad range of psychiatric disorders but these structured interviews lack measures to evaluate ASD. Therefore, this DSM based structured diagnostic interview - that is administered in a similar manner as structured interviews - would complement methods that are often used and can be easily added to the diagnostic interviews. This may improve the efficiency of the assessment, as it is included with screening of other psychiatric conditions.

Our design provides a reasonable estimate of sensitivity (i.e., the probability of our structured interview correctly identifying ASD cases). Remarkably, the sensitivity of the structured interview was extremely high, 94%. Equally remarkable is the finding of a 95% sensitivity of an SRS t-score of 60 or higher (accepted cut-off for a screen of ASD) as an indicator of the gold standard diagnosis. Consistent with these findings, 97% of subjects with our DSM based structured interview diagnosis of ASD also had an SRS t-score in the clinical range (≥ 60). If replicated, these findings would support the utility of a simple to use structured diagnostic instrument based on the defining items for ASD in the DSM-IV to help identify youth with ASD.

Results from our analysis show that the correspondence between our DSM-based structured interview for ASD with the expert clinician assessment was unrelated to the IQ of the subjects in both clinical samples. In addition, our structured interview allowed for the diagnosis of subjects with subthreshold disorders and different definitions of ASDs such as Asperger's Disorder or PDD-NOS, where the ADI-R algorithm score for social impairment may fall below the published clinical cutoff. Thus, our DSM-based structured interview for ASD may be useful and accurate for the assessment of ASD individuals across different cognitive and developmental levels with full and subsyndromal manifestations of these disorders.

Our results must be interpreted in the context of some methodological limitations. Since subjects in this study were referred for ASDs, our results may not generalize to other clinical and non-clinical settings. Because our sample consisted largely of Caucasian subjects, we do not know whether our results will generalize to other ethnic groups. The SRS is validated for youth ages 4 to 18 years but 4% (5/123) of our ASD sample was older than 18 years and received the scale. Although our DSM-based structured interview for ASD was DSM-III-R based, there have been very few changes between the DSM-III-R definitions of ASD and those in DSM-IV (see Table 1). Moreover, we documented a very high correspondence between the DSM-IV based clinician diagnosis of ASD and this instrument. Just as the current version of the structured interview is able to capture both DSM-III-R and DSM-IV diagnostic criteria, a revised version will capture DSM-V measures. As currently proposed, DSM-V criteria for Autism Spectrum Disorders are more narrow and unidimentional. As such, the DSM-based structured interview presented in this paper incorporates the criteria proposed in DSM-V. Coding criteria could also be altered in the future to encompass the changes proposed in the diagnostic criteria for ASD in DSM-V.

## Conclusions

Despite these considerations, our results document the utility of a DSM-based, simple to use and administer, relatively brief structured diagnostic instrument to aid in the identification of youth with ASDs in the clinical setting. If confirmed, these findings would suggest that our DSM-based structured diagnostic instrument for ASD could serve as a rapid and cost-effective assessment instrument to help identify cases likely to meet clinical criteria for ASDs in clinical and non-clinical settings.

## Competing interests (Conflict of Interest)

Gagan Joshi Disclosures: Ethel DuPont Warren Fellowship Award 2005-6; Pilot Research Award from the American Academy of Child and Adolescent Psychiatry 2005; National Institute of Mental Health (Reviewer and member of the NIMH Editorial Board); McNeil Pediatrics (CME sponsored by SynerMed Communications); Bristol Myers Squibb (Site PI for Multi-centered Trial); Glaxo Smith Kline (Site PI for Multi-centered Trial); Shire (Member of national advisory board)

Ronna Fried Disclosures: Dr. Fried has received honoraria from Shire.

Janet Wozniak Disclosures: Dr. Wozniak is the Author of "Is Your Child Bipolar" published May 2008, Bantam Books. Dr. Wozniak is a speaker for McNeil, Primedia/MGH Psychiatry Academy, and is on the Speakers Bureau for Lilly. Dr. Wozniak is on the Advisory Board/Consulting for Pfizer and Shire. Dr. Wozniak receives research support for McNeil, Shire, and Lilly. Dr Wozniak's spouse, John Winkelman, MD, PhD, is on the Speakers Bureau for Boehringer-Ingelheim, Cephalon, GlaxoSmithKline, King, Sanofi-Aventis, Sepracor, and Takeda, is on the Advisory Board/Consulting for Axon Labs, Boehringer-Ingelheim, GlaxoSmithKline, Jazz Pharmaceuticals, Novartis, Neurogen, Novadel Pharma, Pfizer, UCB (Schwarz) Pharma, Sanofi-Aventis, Sepracor, and Takeda, and receives research support from Boehringer-Ingelheim, GlaxoSmithKline, UCB (Schwarz) Pharma, Pizer, and Sepracor.

Jamie Micco Disclosures: Dr. Micco receives funding from the NIMH.

Aude Henin Disclosures: Dr. Henin has received honoraria from Shire, Abbott Laboratories, and AACAP. She receives royalties from Oxford University Press. She has also received honoraria from Reed Medical Education (a company working as a logistics collaborator for the MGH Psychiatry Academy). The education programs conducted by the MGH Psychiatry Academy were, in part, supported though Independent Medical Education grants from pharmaceutical companies, including AstraZeneca, Lilly, McNeil Pediatrics, Shire, Forest Laboratories Inc., Sanofi aventis, Janssen, Bristol-Myers Squibb, and Pfizer.

Robert Doyle Disclosures: Speaker's honoraria- Shire, Novartis, McNeil, Neuroeducational Institute, MGH Academy, and APA. Advisory Boards- Shire, Novartis

Stephen Faraone Disclosures: Dr. Faraone has received consulting fees and has been on Advisory Boards for Eli Lilly and Shire and has received research support from Eli Lilly, Pfizer, Shire and the National Institutes of Health in the past year. In previous years, Dr. Faraone has received consulting fees or has been on Advisory Boards or has been a speaker for the following sources: Shire, McNeil, Janssen, Novartis, Pfizer and Eli Lilly. In previous years he has received research support from Eli Lilly, Shire, Pfizer and the National Institutes of Health.

Joseph Biederman Disclosures: Dr. Biederman is currently receiving research support from Alza, AstraZeneca, Bristol Myers Squibb, Eli Lilly and Co., Janssen Pharmaceuticals Inc., McNeil, Merck, Organon, Otsuka, Shire, NIMH, and NICHD. In 2009, Dr. Joseph Biederman received a speaker's fee from Fundacion Areces, Medice Pharmaceuticals, and the Spanish Child Psychiatry Association. In previous years, Dr. Joseph Biederman received research support, consultation fees, or speaker's fees for/from the following additional sources: Abbott, AstraZeneca, Celltech, Cephalon, Eli Lilly and Co., Esai, Forest, Glaxo, Gliatech, Janssen, McNeil, NARSAD, NIDA, New River, Novartis, Noven, Neurosearch, Pfizer, Pharmacia, The Prechter Foundation, Shire, The Stanley Foundation, UCB Pharma, Inc. and Wyeth.

All other authors report no conflict of interests.

## Authors' contributions

GJ participated in data analysis/interpretation, drafting article, critical revision, and data collection. CP participated in MA: statistics, drafting article, and critical revision. RF participated in concept/design and critical revision. JW participated in concept/design, data analysis/interpretation, and critical revision. JM, AH, RD, MG, MK, JC, and BM all participated in critical revision. SF participated in data analysis/interpretation, and critical revision. JB participated in concept/design, critical revision, and approval of article. All authors read and approved the final manuscript.

## Pre-publication history

The pre-publication history for this paper can be accessed here:

http://www.biomedcentral.com/1471-244X/11/204/prepub
